# Molecular pathways to Parkinson’s disease

**DOI:** 10.1186/1750-1326-7-S1-L13

**Published:** 2012-02-07

**Authors:** Ming Guo

**Affiliations:** 1 Department of Neurology, Department of Molecular & Medical Pharmacology, Brain Research Institute, UCLA David Geffen School of Medicine, LA, California, USA

## Background

Parkinson’s disease (PD) is the second most common neurodegenerative disease. Mutations in PINK1 and PARKIN result in familial forms of PD. We have found that lack of pink1 or parkin in Drosophila results in defects in mitochondrial integrity in multiple tissues. Flies lacking PINK1 or parkin show highly similar, if not identical, phenotypes.

## Results

Genetic studies suggest that PINK1 and parkin function in a common genetic pathway with PINK1 positively regulating parkin. The PINK1/parkin pathway regulates mitochondrial dynamics by positively regulating drp1 and negatively regulating mitofusin. Mammalian cell-based studies suggest that the PINK1/parkin may regulate mitochondrial quality control via mitophagy. How autophagy intersects with mitochondrial dynamics in vivo will be discussed.

